# Supplementary material for: Identification of photoperiod-induced specific miRNAs in the adrenal glands of Sunite sheep (Ovis aries)
Source: Front Vet Sci. 2022 Jul 22;9:888207. doi: 10.3389/fvets.2022.888207 (PMC9354845; doi:10.3389/fvets.2022.888207)
Supplement: Supplementary file 1 [file Data_Sheet_1.ZIP › Supplementary file/miRDeep2 file.zip!/miRDeep2 file/Know_Structure/LP42A1/oar-mir-493.pdf]

miRBase precursor : oar-mir-493
Total read count : 6822
oar-miR-493-5p read count : 4674
oar-miR-493-3p read count : 2147
remaining reads : 1

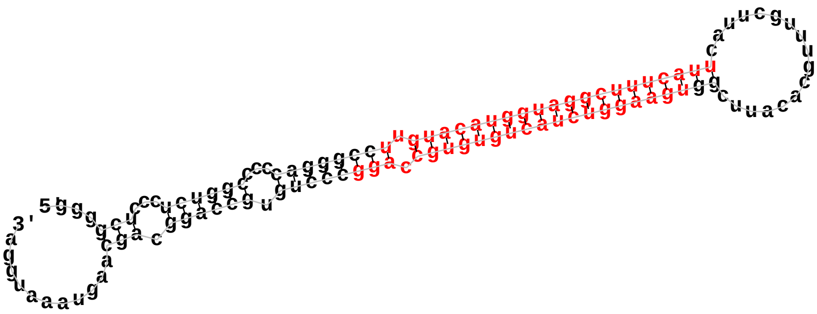

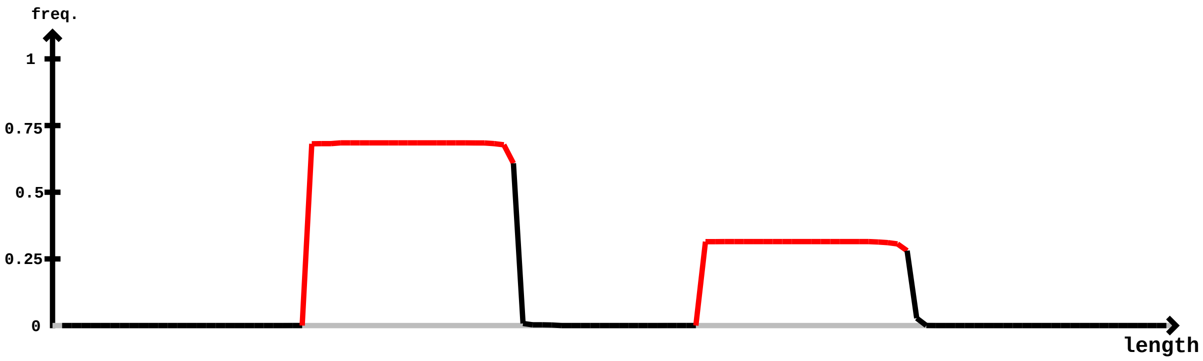

oar-miR-493-5p

oar-miR-493-3p

```
5'- ggggcucccucuggcccccagggccuuguacaugguaggcuuucauucauucguuugcacauucggugaaggucuacugugugccaggcccugugccaggcagcaaguaaaugga   -3'    exp
    ...(((...((((((...((((((((.((((((((((((((((((((.................)))))))))))))))))))).)))))))).)))))).)))...........   reads   mm   sample
    .......................ccuuguacaugguaggcuuuc.......................................................................   1       0    seq
    .........................uuguacaugguaggcuu.........................................................................   2       0    seq
    .........................uuguacaugguaggcuuu........................................................................   1       0    seq
    .........................uuguacaugguaggcuuuc.......................................................................   16      0    seq
    .........................uuguacaugguaggcuuuca......................................................................   30      0    seq
    .........................uuguacaugguaggcuuucau.....................................................................   473     0    seq
    .........................uuguacaugguaggcuuucauu....................................................................   4094    0    seq
    .........................uuguacaugguaggcuuucauuc...................................................................   33      0    seq
    ..........................uguacaugguaggcuuucauu....................................................................   3       0    seq
    ..........................uguacaugguaggcuuucauuc...................................................................   1       0    seq
    ............................uacaugguaggcuuucauuc...................................................................   1       0    seq
    ............................uacaugguaggcuuucauucau.................................................................   5       0    seq
    ............................uacaugguaggcuuucauucauu................................................................   14      0    seq
    ..............................................................ucggugaaggucuacugugugc...............................   1       0    seq
    ..................................................................ugaaggucuacugugugU...............................   1       1    seq
    ..................................................................ugaaggucuacugugugG...............................   1       1    seq
    ..................................................................ugaaggucuacAgugugc...............................   1       1    seq
    ..................................................................ugaaggucuacugugugc...............................   7       0    seq
    ..................................................................ugaaggucuacugugugcc..............................   16      0    seq
    ..................................................................ugaaggucuacugugugcca.............................   33      0    seq
    ..................................................................ugaaggucuGcugugugccag............................   2       1    seq
    ..................................................................ugaaggucuacugugugccag............................   169     0    seq
    ..................................................................ugaaggCcuacugugugccag............................   1       1    seq
    ..................................................................ugaagAucuacugugugccag............................   1       1    seq
    ..................................................................ugaaggucuacuAugugccag............................   1       1    seq
    ..................................................................ugaaggucuGcugugugccagg...........................   5       1    seq
    ..................................................................ugaaggCcuacugugugccagg...........................   9       1    seq
    ..................................................................ugaaggucuacugugugccagg...........................   1708    0    seq
    ..................................................................ugaagguUuacugugugccagg...........................   1       1    seq
    ..................................................................ugaaggucuacuAugugccagg...........................   2       1    seq
    ..................................................................ugaaggCcuacugugugccaggc..........................   1       1    seq
    ..................................................................ugaaggucuacugugugccaggc..........................   186     0    seq
    ....................................................................aaggucuacugugugccaggcc.........................   2       0    seq
```
